# Expert assessment of the impact of ship-strikes on cetacean welfare using the Welfare Assessment Tool for Wild Cetaceans

**DOI:** 10.1017/awf.2023.7

**Published:** 2023-02-23

**Authors:** Francesca Rae, Christine Nicol, Mark P Simmonds

**Affiliations:** 1 Bristol Veterinary School, Langford House, Dolberry, Bristol BS40 5DU, UK; 2 The Royal Veterinary College, Hawkshead Lane, North Mymms, Hatfield AL7 9TA, Herts, UK

**Keywords:** animal welfare, five domains model, humpback whale, ship-strike, WATWC, wild animal welfare

## Abstract

Human activities are increasingly impacting our oceans and the focus tends to be on their environmental impacts, rather than consequences for animal welfare. Global shipping density has quadrupled since 1992. Unsurprisingly, increased levels of vessel collisions with cetaceans have followed this global expansion of shipping. This paper is the first to attempt to consider the severity of ship-strike on individual whale welfare. The methodology of the ‘Welfare Assessment Tool for Wild Cetaceans’ (WATWC) was used, which is itself based upon the Five Domains model. Expert opinion was sought on six hypothetical but realistic case studies involving humpback whales (*Megaptera novaeangliae*) struck by ships. Twenty-nine experts in the cetacean and welfare sector took part. They were split into two groups; Group 1 first assessed a case we judged to be the least severe and Group 2 first assessed the most severe. Both groups then additionally assessed the same four further cases. This was to investigate whether the severity of the first case influenced judgements regarding subsequent cases (i.e. expert judgements were relative) or not (i.e. judgements were absolute). No significant difference between the two groups of assessors was found; therefore, the hypothesis of relative scoring was rejected. Experts judged whales may suffer some level (>1) of overall (Domain 5) harm for the rest of their lives following a ship-strike incident. Health, closely followed by Behaviour were found to be the welfare aspects most affected by ship-strikes. Overall, the WATWC shows a robust potential to aid decision-making on wild cetacean welfare.

## Introduction

Ship-strike is recognised as one of the greatest sources of human-caused mortality for whales (Redfern *et al*. 2020). A four-fold global growth in shipping traffic between 1992 and 2012 was identified by ship-traffic analysis, with growth in all ocean basins (Tournadre 2014). With a continuing growth of shipping traffic and average vessel speeds increasing, ship-strike has become an increasingly important and well-recognised problem (Cates *et al*. 2017; Ritter & Panigada 2018; Rockwood *et al*. 2021). For example, it has recently been estimated that ten humpback whales (*Megaptera novaeangliae*) are killed annually by ship-strikes on the Californian coast alone (Rockwood *et al*. 2021). Some whale populations are showing recovery towards pre-whaling exploitation levels (Cooke 2018). The combination of growing whale populations and increased shipping means that ship-strikes will likely become more of an issue and an increasing welfare concern (Smith *et al*. 2020).

It has been argued that where pursuit of human interests adversely affects wild animal welfare, efforts should be undertaken to change practices to prevent or minimise welfare consequences (Kirkwood 2013). Yet, to date, most international wildlife law is concerned with the *conservation* of species and usually ignores the *welfare* of individual animals (Scholtz 2017). This seems to be true even where many individuals are suffering poor welfare. However, the emergence of a new discipline — ‘Conservation Welfare’ — now offers the potential to integrate the expertise of scientists from both conservation and welfare backgrounds and move towards the concept of ‘feelings *and* fitness’ rather than ‘feelings *or* fitness’ (Beausoleil *et al*. 2018). Ship-strike is an issue where conservation welfare science should be applied and where the scientific assessment of the welfare consequences of ship-strike may inform decisions to take action to reduce impacts (Papastavrou *et al*. 2017). However, a paucity of published scientific papers acknowledge the welfare consequences caused by these collisions.

There are many difficulties inherent in the assessment of wild animal welfare (Beausoleil & Mellor 2015). Collecting the necessary data from free-living individuals is not always possible and arguably this is especially the case for highly mobile, sea-dwelling animals. Measuring glucocorticoid hormones has however been applied and is an accepted approach to measure physiological stress (Rolland *et al*. 2017). Rolland *et al*. reported substantial elevation of faecal glucocorticoid concentrations in North Atlantic right whales (Eubaelania glacialis) entangled in fishing gear, but no increase in right whales killed ‘quickly’ by ship-strikes. However, this study did not assess the stress levels of whales injured by a ship-strike or of those that died sometime after a strike. Such studies are only possible in well-studied whale populations, and are of most use with identifiable individuals and longitudinal life history data (Rolland *et al*. 2017).

Therefore, noting that expert opinion is currently the best (or only) method available to develop a picture of the overall welfare impact of a scenario (McGreevy *et al*. 2018), this study used the ‘Welfare Assessment Tool for Wild Cetaceans’ (WATWC). The WATWC is based on the Five Domains approach and provides a framework to seek expert opinions (Nicol *et al*. 2020). This study is only the WATWC’s third formal deployment for cetaceans (Nicol *et al*. 2020; King *et al*. 2021), although it has recently been adapted for use eliciting opinion about penguin welfare (Freire *et al*. 2021).

The Five Domains model is a well-accepted tool that has been applied to both captive and domestic animal welfare (Beausoleil & Mellor 2015), but only recently has it been used to guide assessments for wild animals (Beausoleil *et al*. 2016; Nicol *et al*. 2020). The model provides a systematic method for identifying compromise in four physical/functional domains (Nutrition, Environment, Health, Behaviour) and one mental domain which reflects the animal’s overall welfare state (Affective State) (Mellor & Beausoleil 2015; McGreevy *et al*. 2018). It acts primarily as a framework, helping to ensure that assessors consider a wide range of influences and their possible effects.

The primary aim of this study was to assess the likely severity of ship-strike on the welfare of humpback whales. Six case studies were compiled involving ship-strike incidents with humpback whales in the Indian Ocean/southern hemisphere where the largest increase in shipping traffic between 1993 and 2012 was seen (Tournadre 2014). Humpback populations here are classed as being of ‘Least Concern’ on the International Union for Conservation of Nature (IUCN) (Cooke 2018) allowing experts to answer from a welfare perspective without being overly influenced by population viability. It was also deemed important to use the same species throughout the cases so results would not be affected by species differences.

Experts were asked to grade the impacts to each domain out of ten (ten being most severe). Expert assessments were based on: (i) the evidence available; and (ii) their knowledge and experiences — making analogies and generalising based on evidence from other species or contexts, e.g. captive cetaceans, better-studied whale populations such as the North Atlantic right whale, or free-living mammals of other (more easily studied) species.

The extent to which humans exhibit absolute versus relative preferences in decision-making is an active field of research (e.g. Teodorescu *et al*. 2015) and was a novel aspect considered in our study. In many circumstances, the choices of humans (e.g. Soltani *et al*. 2012) and other animals (Bateson *et al*. 2004) are influenced by the composition of the choice set (or context) under consideration (Bateson 2004). Choices do not always depend on the absolute properties of each alternative but are affected by the nature of the alternatives that are available. Judgements may be especially influenced by the information available as a starting point (anchoring bias) (Chapman & Johnson 1994). Previous work using the Five Domains model mentioned but did not analyse the effects that absolute vs relative evaluation might be having on their results. Assessors have used the Five Domains model framework to score the impacts of various procedures within a certain context and it is possible that opinions on a certain procedure within the context may be influenced by the other procedures presented. For example, in a paper on the welfare consequences of different husbandry practices that impact horses, McGreevy *et al*. (2018) explicitly suggested that a given score in the context of equine care may not have been equivalent to the same score provided in the context of equine surgery. Our current study therefore aimed to investigate empirically whether assessors evaluated welfare consequences relatively or absolutely by adjusting the severity of the initial case assessed. It was hypothesised that participants would rate in a relative fashion. Our secondary aim was therefore to test this hypothesis and consider its implications. If confirmed, results would not be able to be compared between studies, as scores would depend on the context and severity of the other cases presented.

## Materials and methods

### Ethical approval

Ethical approval was granted by the University of Bristol’s Human Ethics Board – the Faculty of Health Science Student Research Ethics Committee (HSSREC). The Reference ID was 103503. A signed consent form was completed by each participant before they took part in the survey.

### Developing the case studies

We used the Five Domains model as a framework to assess the welfare implications of six case studies based on real-life scenarios. The cases were developed after completing a thorough literature search on ship-strike injuries. Using the database Web of Science, 55 papers were found when using the search terms (cetacean OR dolphin* OR whale*) AND (ship OR boat OR vessel) AND (strike OR collision) AND (injury). A literature search without the term ‘injury’ was also carried out. Two hundred and twenty papers were available and any additional papers discussing wounds were considered during the development of the case studies. Due to the relative lack of research on such injuries, all cetacean species were included in the search. From the source material gained, five realistic and contrasting cases were developed based on real examples (Table 1; Moore *et al*. 2013; Aschettino *et al*. 2020; Squires 2020). As cases needed to be comparable with each other, three of the cases involved injuries to the whale’s tail and all featured a humpback whale.

**Table 1.** The case studies. The ‘instant death case’ was Case 5a in the original document sent to assessors. It was considered as a separate case for analysis

**Case 1A** – WATWC for juvenile with laminar incising wounds	A 2 y.o. juvenile humpback whale was spotted in the Antarctic, its feeding ground, with a small, sublethal propeller incision (extending just into the dermis, not reaching the blubber) on its dorsal torso. The injury showed second intention healing and no sign of infection. The accident was thought to have occurred over a month ago. The individual was no longer maternally dependent.
**Case 1B** – WATWC for injured juvenile whale	A 2 y.o. juvenile humpback whale was spotted in the Antarctic, its feeding ground, with severely impaired locomotion. Focally extensive subcutaneous haemorrhage was visible, and it was diagnosed as having a shattered lumbar vertebra and multiple fractures to the left transverse processes of the thoracic vertebrae due to collision with the hull of a ship. The accident was thought to have occurred within the last week. The individual was no longer maternally dependent.
**Case 2** – WATWC for injured 20 y.o. female	A 20 y.o. female humpback whale was struck by the propeller of a ship moving at 12 knots off the coast of Mozambique. The whale had been photographed on 15^th^ July with no apparent injuries or health concerns. When spotted again on 15^th^ August (a month later), there was a laceration to its left tail fluke which had removed the tip of the tail leading to a unilateral amputation. The wound had become necrotic and the accident was thought to have occurred two weeks ago.
**Case 3** – WATWC for injured calf	A 4 month old, female, humpback whale calf was struck by the propeller of a ship moving at 12 knots off the coast of Mozambique. The individual was still maternally dependent. The whale calf had been photographed on 15^th^ July with no apparent injuries or health concerns. When spotted again on 15^th^ August (a month later), there was a laceration to its left tail fluke which had removed the tip of the tail leading to a unilateral amputation. The wound had become necrotic and the accident was thought to have occurred two weeks ago. Its mother was still present, healthy and accompanying the calf.
**Case 4** – WATWC for tailless 20 y.o. humpback whale	A 20 y.o. female humpback whale was struck by the propeller of a ship moving at 12 knots off the coast of Mozambique. The whale was spotted for the first time on 15^th^ August with a fluke amputation at the mid-peduncle (leaving it completely tail-less). The tissue was visibly necrotic. The accident was thought to have occurred within the last week.
**Case 5** – WATWC for the orphaned calf, 2 days on from the accident	A 20 y.o. female humpback whale collided with a ship’s hull off the coast of Mozambique in September. The ship was going at 18 knots and the blunt force trauma led to immediate death for the individual. This was observed by a mariner onboard the ship who reported the accident and saw the whale immediately sinking, indicating death. The individual was thought to be a whale that had been spotted a month prior with no apparent injuries or health issues and with a 4 month old dependent calf in tow. At the time of the injury the mariner reported seeing the calf swim away, unscathed. Humpback calves nurse from and stay near to their mothers for up to one year before weaning. Therefore, the calf was still maternally dependent at the time of the accident. Calves are not believed to maintain long-term associations with their mothers.
**Instant death case** – WATWC for the mother whale who died immediately	A 20 y.o. female humpback whale collided with a ship’s hull off the coast of Mozambique in September. The ship was going at 18 knots and the blunt force trauma led to immediate death for the individual. This was observed by a mariner onboard the ship who reported the accident and saw the whale immediately sinking, indicating death. The individual was thought to be a whale that had been spotted a month prior with no apparent injuries or health issues and with a 4-month-old dependent calf in tow. At the time of the injury the mariner reported seeing the calf swim away, unscathed. Humpback calves nurse from and stay near to their mothers for up to one year before weaning. Therefore, the calf was still maternally dependent at the time of the accident. Calves are not believed to maintain long-term associations with their mothers.

Cases were carefully written with a neutral stance, e.g. ‘the whale is going to survive for three more months, it will not be able to feed in its last week’ and not ‘the whale will likely starve to death slowly.’ Care was taken to avoid any leading or suggestive information. The preliminary texts describing the cases were reviewed by two experts and then amended accordingly.

To determine whether experts were making absolute or relative judgements between different cases, assessors were divided into two groups and initially presented with one of two initial cases (Case 1A or 1B), but without prior knowledge of our intention to examine how this influenced scoring pattern. 1A was written to have, anticipated based on the available literature, the least serious welfare consequences of any of the cases presented, and 1B the most severe (Table 1). Therefore, in accordance with our hypothesis that assessors would score cases relative to each other, we predicted that the participants sent Case 1A would score the subsequent cases more severely than the participants who received Case 1B. To assess this, Mann-Whitney *U* tests were carried out to see whether exposure to a different initial case influenced scoring on Cases 2 to 5 which were identical for both groups.

Experts also considered an additional scenario relating to the immediate death of a whale due to blunt force trauma sustained during a collision. Due to differing interpretations of ‘immediate’ this case was analysed separately and not included as part of the main study (Table 1).

### The scoring sheet

**Figure 1.**
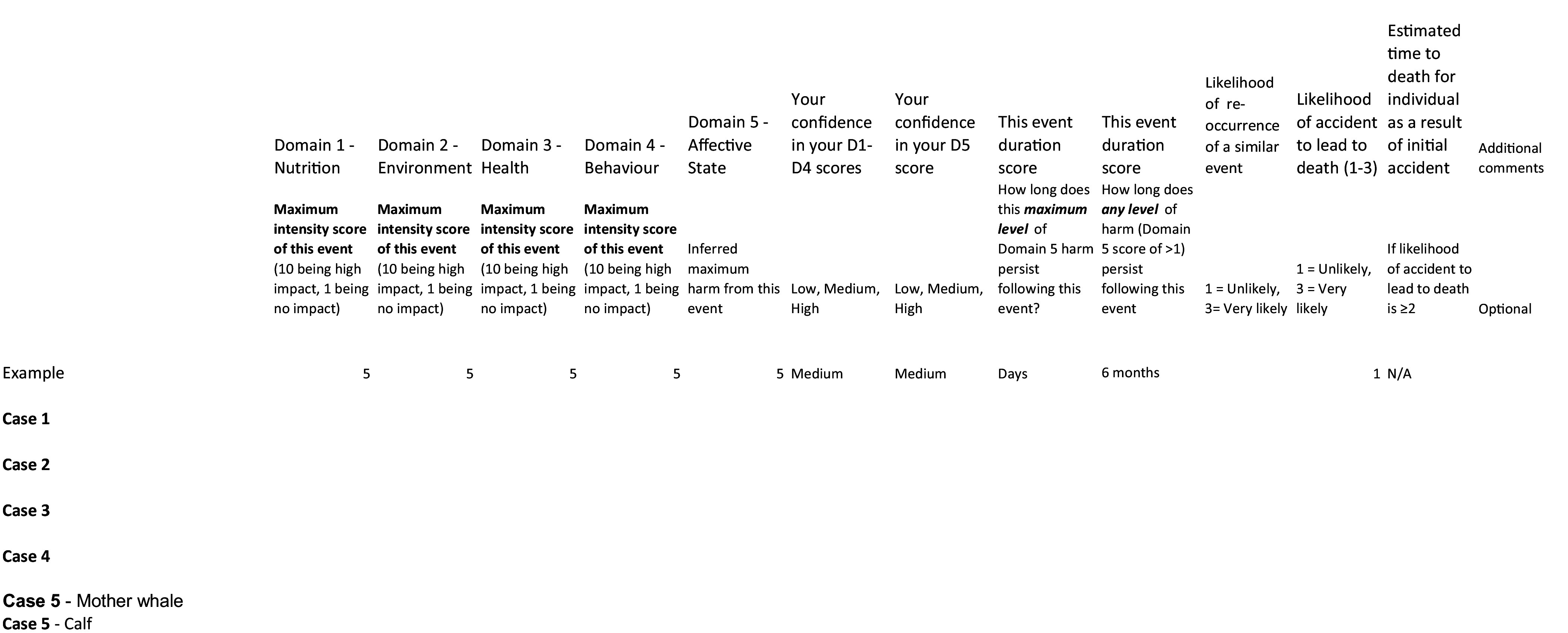
The WATWC scoring sheet sent to assessors.

The original WATWC scoring sheet (Nicol *et al*. 2020) was adjusted to be more specific to ship-strike cases. The following sections remained unchanged: Domains 1–5 (Nutrition = D1, Environment = D2, Health = D3, Behaviour = D4, Affective State = D5); the ‘Confidence in Your Scores’; and the ‘Event Duration Score.’ Three new headings were added: ‘Likelihood of Re-occurrence of a Similar Event’; ‘Likelihood of Accident to Lead to Death’; and ‘Estimated Time to Death for Individual as a Result of Initial Accident.’ These were changed because the original tool was designed to assess the impact of chronic threats to welfare that might occur throughout a whale’s lifespan rather than a single event, such as ship-strike. A final column was added for additional comments. Figure 1 shows the final refined version of the WATWC scoring sheet sent to assessors.

### Expert list and engagement

A list of relevant experts was compiled from a literature search and encompassed animal welfare and/or cetacean experts. The experts fell into one or more of the following categories: cetacean scientist; welfare specialist; or wildlife veterinarian. The final list comprised 266 experts who received an email with an explanatory cover letter and six documents. The documents consisted of the HSSREC consent form, an accompanying Participant Information Sheet, pages detailing the five case studies (either with Case 1A or Case 1B), an optional background information sheet and a table detailing the Five Domains model specific to cetacean welfare (as developed by Nicol *et al*. 2020). Case 1A was sent to 126 experts and Case 1B to 140 experts. Cetacean scientists and welfare specialists were spread evenly between the two groups. Experts had up to four weeks to complete the tool independently and without conferring.

Of the 266 experts contacted, 37 replied by email, 29 agreed to take part and all returned their score-sheets while eight declined due to perceived insufficient knowledge of the topic. The 29 participants were sent a follow-up email asking them an open question about their general area of background expertise. From the 24 responses received (see Supplementary Material), the authors developed seven initial categories that attempted to encompass the range of expert backgrounds but, due to low numbers in some categories (e.g. there were only two veterinarians among the participants), these were collapsed down to two broad categories for subsequent analysis (‘cetacean specialist’ with primary expertise in cetaceans, often alongside other specialist interests, or ‘welfare expert’ with primary expertise in general animal health, welfare or behaviour). For the five participants who did not volunteer information about their background expertise, the authors reviewed publicly available information about their publications, research projects and biographical information before allocating them to the cetacean specialist or welfare expert category. Overall, there were 16 cetacean specialists (five received Case 1A and eleven received Case 1B) and 13 welfare experts (ten received Case 1A and three received Case 1B).

### Statistical analysis

**Figure 2.**
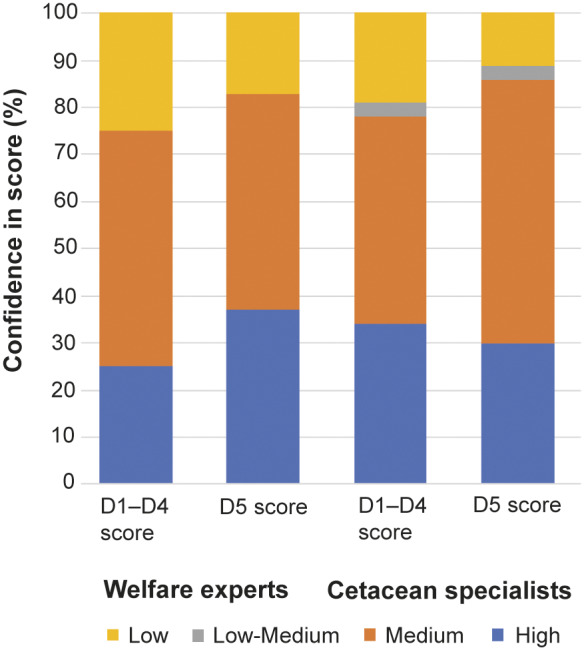
Spread of confidence in domain scores across the two groups of experts. Experts were grouped into these two broad categories and confidence scores were combined for Cases 2–5.

**Figure 3.**
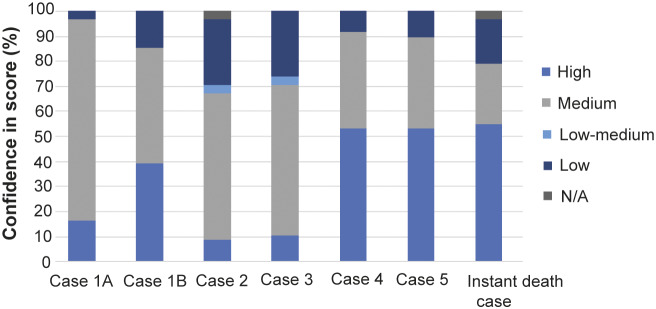
Spread of confidence across the cases. D1–D4 and D5 confidences have been combined. A single assessor did not see the purpose of filling out Case 5a as the whale was dead and so confidence score was assigned as ‘N/A.’

The percentage of assessors expressing confidence in their scores was compared informally by assessor background (Figure 2) and by case (Figure 3). A Wilcoxon signed rank test was carried out to compare the confidence of each assessor for their average D1 to 4 scores (across all cases) vs their D5 scores (across all cases). This was done by converting the categories Low, Low-medium, Medium, and High into an ordinal scale (1, 2, 3, 4).

The average case scores were compared using Friedman repeated-measures analysis, followed by pair-wise comparisons. Spearman rank correlation was used to examine associations between scores across different domains. Mann-Whitney *U* tests were used to compare the Case 2 to 5 scores given by assessors from Group A (initial case least severe) and B (initial case most severe).

## Results

### Case summaries

#### Case 1A: Laminar incising wound from ship’s propeller. Two year old calf

This, the least severe case, had a median affective state score of three. Experts thought the whale would likely recover with minimal long-term consequences.

Two assessors explained their scores. The first noted that “this likely caused a good deal of pain for several days. Some non-visible damage could have occurred, and the immune system may be weakened for an extended period in order to fight any infections until healing is complete.” The other commented “the pain associated with this is minor, it decreases and there are no long-term consequences.”

#### Case 1B: Blunt force trauma accident. Broken vertebrae. Two year old calf

As the median affective state score was nine (ranging from six to ten), the experts considered that this event had serious consequences on the individual. D2 had the lowest average, but one welfare expert scored it ten believing the whale would not be able to migrate. Another assessor said they had seen a real-life case where a similar accident had led to death of an adult right whale within a week. One assessor explained their D2 score (of seven) was due to an increased predatory threat, and their D4 score (of four) was due to impaired sleep/rest. Another stated the severity of the injury would impact feeding and cause a loss of Body Condition Score.

#### Case 2: Sharp trauma accident with a ship’s propeller. Unilateral amputation. Twenty year old

Domain 5 scores ranged from two to ten for this case with a median of six. Nineteen out of 28 assessors (68%) gave a score of six or above, indicating moderate severity. Although for both groups of assessors Domain 5 received the same median score, Group B assessors had a higher proportion of scores below six, with five out of 14 giving a score of between two and five.

Some level of uncertainty was expressed in the additional comments section with six assessors saying the wound may become infected and lead to septicaemia, which could progress to death. One of these said a systemic bacterial infection was unlikely in seawater. Another said they had seen many humpback whales with similar fluke-tip amputations survive for many years but said some level of D5 harm would persist for ‘years’. A cetacean expert had a similar view saying “with only the tip of the tail missing, the whale would still be able to function as normal. There may be slight increased risk of slower movements and more difficulty communicating with its tail fluke, however being 20 years old, she will likely quickly adapt and continue life as normal.”

Another expressed uncertainty as to whether it was just the tip of the left fluke missing or the whole fluke and suggested use of a diagram or photograph.

#### Case 3: Sharp trauma accident with a ship’s propeller. Unilateral amputation. Four month-old calf

Domain 5 scores ranged from two to nine with a median of 6.5. Three assessors mentioned the increased predatory threat to the calf and two mentioned the calf would be vulnerable during migration, with one expert stating there would be an increased likelihood of death, as it is critical the calf gains weight and size in order to make its first migration keeping up with its mother’s speed. Four experts highlighted the importance of the mother being around to protect the calf and provide nutrition until it weans.

Assessors thought this was ‘likely’ to lead to death whereas, in Case 2, it was considered ‘unlikely’ to lead to death. This again highlights the increased severity of ship-strike to calves.

#### Case 4: Sharp trauma accident with ship’s propeller. Complete tail amputation. Twenty year old

The median Domain 5 score was 9.5 indicating very severe welfare consequences. One hundred percent of assessors gave a score of seven or above, with the majority (n = 14) giving a score of ten. Nutrition was the most affected with 23 giving it a score of nine or above, closely followed by Behaviour. It has been argued that impacts in these domains are usually more relevant to the overall welfare of an individual than high scores in the Health domain (Beausoleil & Mellor 2015).

This case had the highest ‘agreed upon’ severity (i.e. the highest D5 score combined with the highest level of confidence and smallest range in scores).

It proved ‘likely’ to lead to death with one expert explaining it depends on the ability of the whale to manoeuvre and forage. One said time to death would depend upon how long it could survive on its body reserves. Similarly, another said the whale would die two months after the accident if the wound became infected, otherwise it could survive for 9–12 months or more before dying (of starvation). It would be unlikely the individual would be able to migrate to feeding grounds or dive properly.

#### Case 5: Impact on calf after the death of mother due to blunt force trauma caused by impact with ship’s hull

Domain 5 scores ranged from seven to ten with 68% of assessors scoring ten, therefore it had the most severe consequences for Domain 5 out of all the cases.

Two assessors allowed for the slight possibility that the calf may survive. A ‘cetacean and welfare expert’ stated there to be a very slight possibility of the calf being adopted by another lactating female. Five experts said the calf would either be predated upon without protection from its mother or starve to death (still being 100% dependent on its mother for nutrition).

#### Instant death case

This case showed the widest variety in D1–D5 scores, ranging from zero to ten. Seventeen assessors gave a D5 score of eight or above and nine (five ‘welfare experts’ and three ‘cetacean experts’) a score of three or below. One explained their D5 score of two was due to short-term surprise/fear just before shock kicked in.

Two experts left the whole case blank and one, D1 and D4 blank, stating it impossible to score the welfare of a dead animal. One said “I could have scored all of these as zero or ten, because it is unlikely the whale knew much about this incident, but it has been killed so I wanted to express the impact this had. Maybe this scale is inappropriate here.” One added, “this could be considered to be the most severe impact to welfare because of loss of life or the least severe because, although the life of this animal was lost, very little pain and suffering occurred; even if death was ‘immediate’ it is likely the animal was in extreme pain, temporarily.” Two experts said that although it is stated the animal died immediately, a large animal would take minutes to die from blunt trauma.

### Confidence in scores

Overall, across Cases 2 to 5 which were assessed by all participants, 23% had ‘low’ confidence in their answers relating to Domains 1 to 4, and 15% had ‘low’ confidence in answers relating to Domain 5. Confidence in D5 was significantly higher than confidence in average D1 to D4 score (Wilcoxon test statistic: 320, n = 115; *P* < 0.06).

Figure 2 shows how category of expertise was associated with self-generated confidence score. ‘Welfare experts’ had lower confidence in their D1–D4 scores than ‘cetacean specialists.’

The confidence scores for each case can be compared in Figure 3. Assessors expressed the greatest level of ‘medium’ confidence for Case 1A out of all the cases (80%). However, the level of ‘high’ confidence expressed was considerably lower (17%) than that expressed for Cases 1B (39%), 4 (53%), 5 (53%) and ‘instant death case’ (55%). Cases 2 and 3 saw the most uncertainty with the highest levels of ‘low’ or ‘low-medium’ confidences expressed, whilst Cases 4 and 5 had the highest level of certainty (54% had ‘high’ confidence in their answers, and 91 and 89%, respectively, expressed ‘medium’ or ‘high’ confidence combined). These also had the highest median affective state (D5) scores.

Seven out of 29 experts found the exercise ‘tricky’ or ‘hard.’ Two said there was a lot of ‘guessing’ in their answers. One animal welfare expert with a specific focus on dairy cattle said they did not feel qualified enough to assess the welfare of cetaceans, but nonetheless returned the score-sheets. Two assessors said they found the welfare assessment tool subjective and/or struggled with the concept.

### Participant assessment of case severity

The overall pattern of scoring across the cases that were assessed by all participants is summarised by the median scores shown in Table 2. Domain 5 scores did not differ between Cases 2 and 3 (*P* = 0.64), or between Cases 4 and 5 (*P* = 0.72), but scores for Cases 2 and 3 were significantly lower than for Cases 4 and 5 across all comparisons (*P* < 0.001). This pattern of results was unaffected when Bonferroni corrections for multiple comparison were applied.

**Table 2.** Median scores for Domains 1–5 across the cases. Group A and Group B assessors’ scores have been combined for Case 2–5

	Domain 1 - Nutrition	Domain 2 - Environment	Domain 3 - Health	Domain 4 - Behaviour	Domain 5 - Affective State
Case 1A	2	1.3	3	2.3	3
Case 1B	8.5	6	9	9	9
Case 2	5	4.5	6	6	6
Case 3	3	5	7	5	6.5
Case 4	9	7.5	9	9	9.5
Case 5	1	1	10	10	10
Instant death case	10	8	8	10	10

We next considered how assessors may have arrived at their Domain 5 scores for overall affective state. First, we noted that D5 scores given by assessors were more strongly correlated with their scores for Domains 3 (Health) and 4 (Behaviour) than with their scores for Domain 1 (Nutrition) or 2 (Environment). The correlation coefficients for each of these measurable domains against inferred affective state are shown for each case in Table 3. The lack of significance for Cases 1A and 1B reflects low sample size, with half the participants assessing each of these cases.

**Table 3.** Correlation coefficients (Spearman Rho) for each of the four measurable domains

Scenario	D1 Nutrition	D2 Environment	D3 Health	D4 Behaviour
1a	0.355	0.5	0.23	0.403
1b	0.435	0.06	0.546*	0.527
2	0.464*	0.380*	0.768**	0.577**
3	0.449*	0.275	0.732**	0.678**
4	0.082	0.247	0.486*	0.528**
5 (calf)	0.344	−0.02	0.256	0.503**

* Indicates a correlation significant at p < 0.05.

** Indicates a correlation significant at p < 0.01.

**Figure 4.**
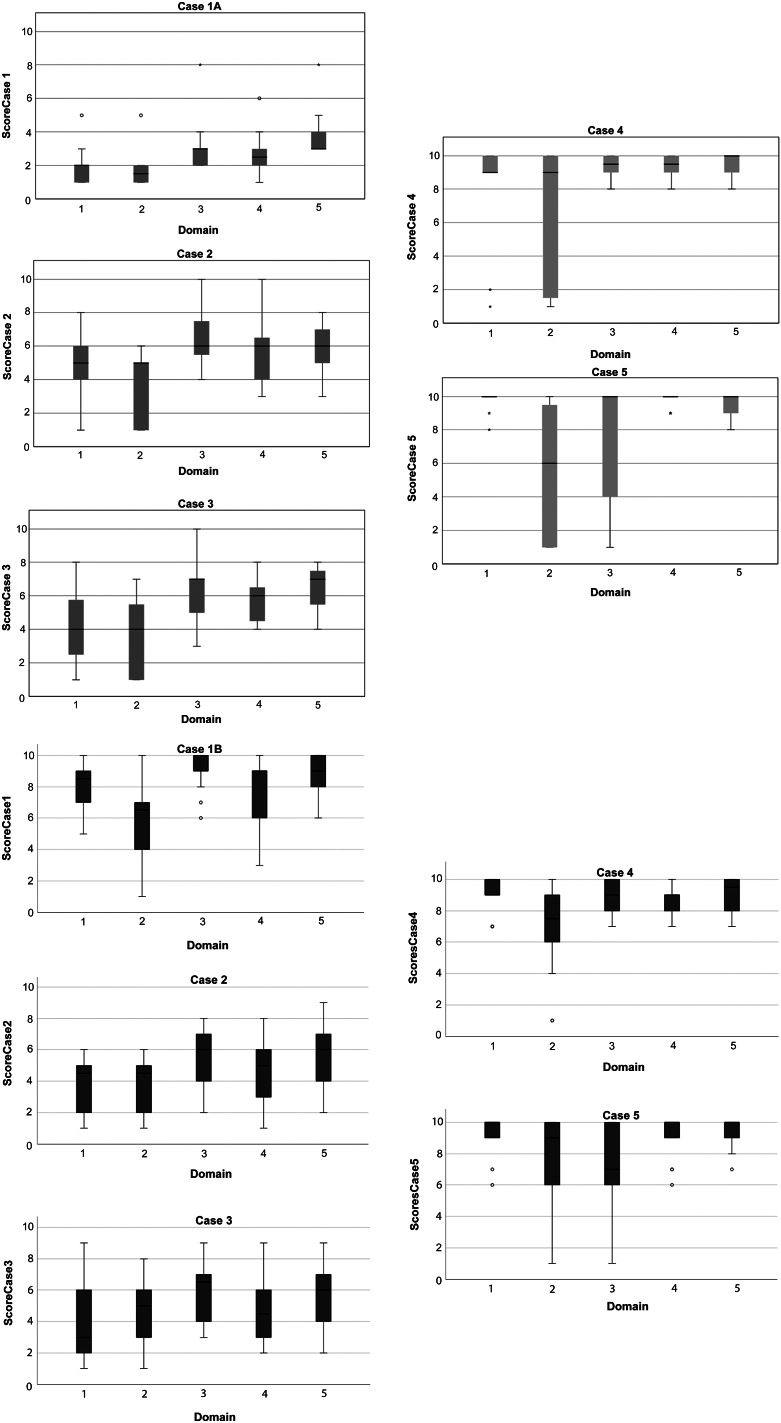
Boxplots for Cases 1–5 with each Domain score shown with the results for Group A, who received an initial less severe case to evaluate (Case 1A) (a) and the results for Group B (b) who received an initial severe case to evaluate (Case 1B).

Generally, assessors considered that ship-strikes had more effect on Domain 3 (Health) than the other domains (Table 2). As expected, Domain 5 scored equal to or higher than any of the D1–D4 scores, apart from in Case 5A (renamed the ‘Instant death case’) where one assessor (a welfare expert) gave D1–D4 scores of ten and D5 a score of two. Scores given for Domain 2 (Environment) were generally more variable than for the other Domains (see Figure 4).

The event duration scores varied considerably between assessors. Experts were asked; ‘How long does this *maximum level* of Domain 5 harm persist following this event?’ and ‘How long does *any level* of harm (Domain 5 score of > 1) persist following this event.’ Answers were broad even within cases. Case 3 has answers from ‘days’ to ‘years’ for maximum level of Domain 5 harm, with three weeks being the modal answer (28% of answers). ‘Lifetime’ or ‘until death’ was the modal answer across all the cases combined for how long ‘any level’ of Domain 5 harm would persist. The results did highlight a Domain 5 harm of > 1 may persist for months to years in even the least severe cases, with ‘months’ being the modal D5 harm duration for Case 1A.

### Absolute versus relative preference

**Figure 5.**
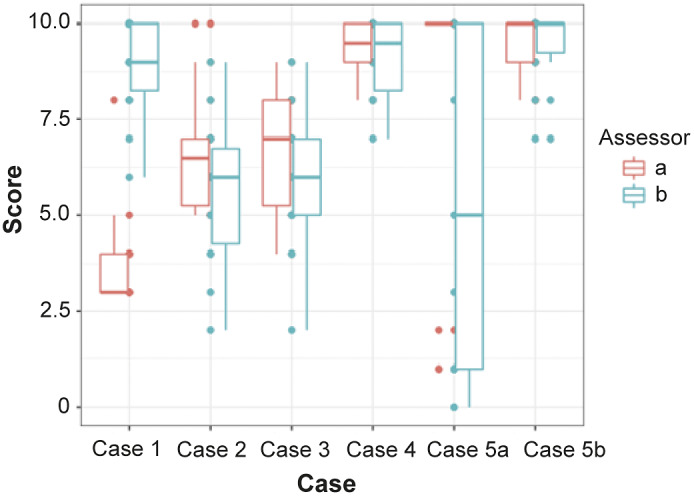
Domain 5 scores for all cases showing the different scores between the two groups of assessors.

Figure 5 shows the Domain 5 scores given by Group A assessors and Group B assessors plotted as boxplots with whiskers. Group A assessors scored Case 1A very differently from Group B assessors’ scores for Case 1B. The Mann-Whitney *U* tests showed no significant differences between Group A and Group B (Case 2, *U* = 85; *P* = 0.057; Case 3, *U* = 75; *P* = 0.201; Case 4, *U* = 96.5; *P* = 0.714; Case 5, *U* = 108; *P* = 0.914).

## Discussion

### Cases and domains

Domain 2 generally had the largest range in scores as demonstrated by the boxplots in Figure 4. This may be due to ‘environment’ being harder to interpret than the other domains and having a greater variability of potential impacts. For example, ‘welfare risk’ and ‘welfare impact’ could have been confused (e.g. an increased *risk* of predation, does not mean the individual is actually *impacted* by predation). This increased risk of predation would be described as a welfare alerting factor (Harvey *et al*. 2020). These prompt further monitoring, but cannot be used in the overall assessment of welfare unless the individual has actually been seen to be affected by it (e.g. if the whale had a shark bite/teeth marks). Harvey *et al*. (2020) explain the importance of only using animal-based indices (i.e. externally observable or internally measurable factors, such as Body Condition Score of the animal) when assessing overall welfare status. Therefore, high scores in Domain 2 may be misleading. It was hoped the Five Domains model for wild cetaceans would reduce the difficulty in interpretation by outlining factors that may contribute to each domain. After this research was conducted, a new paper was published detailing an updated Five Domains model, whereby Domain 4 was classed as ‘Behavioural interactions’ (Mellor *et al*. 2020). It is possible that this updated version might have had an impact on assessors scores for this domain.

The general increase between D1–D4 and D5 confidence, supports the intention of the model to encourage assessors to integrate all previous scores into their overall Domain 5 assessment. It is possible that scores in Domain 5 are also more reliable (repeatable) than scores in other domains, though this remains to be investigated.

Case 3 received a slightly higher Domain 5 score than Case 2 which was the same case but with a 20 year old whale instead of a calf. This implies the severity of such an injury is more serious for calves compared to adults, and previous research suggests ships are more likely to collide with calves or juveniles (Lammers *et al*. 2013).

Cases 4 and 5 had the highest proportion of high assessor confidence scores, along with the highest median affective state (D5) scores. This suggests the more ‘severe’ the case, the more certainty assessors had in their scores. One reason for this may be that the outcome of major injuries is more restricted, whereas more minor injuries might have more possible outcomes. This said, the ‘instant death case’ showed the widest variety in D1–D5 scores yet assessors scored with the highest confidence of all (57% expressing high confidence in their scores), perhaps indicating that death is easier to assess than welfare. The scores received for this case were extreme (either zero or ten depending on whether assessors believed that death really had been instantaneous). We note that it has been reported that 100% of collisions with ships travelling over 18 knots are fatal to the whale (Van Waerebeek *et al*. 2007). One assessor (a welfare expert) scored ten for D1 through to D4 theorising if the incident was lethal this must have maximum impact on those domains. However, a score of two was given for D5 due to “short term surprise/fear just before the shock happened; but if we can actually be sure that instant death happens, then there should be limited impact on affective state.” Logically, Domain 5 scores should never be less than the highest score given across D1–D4 therefore this shows the difficulty of using this method to assess instant death. It highlights the potential usefulness of other modified Five Domains models such as that by Sharp and Saunders (2011) and Beausoleil *et al*. (2016) which separate the Five Domains model into Part A (impact on domains before action leading to death) and Part B (mode of death). Nine out of 29 assessors had trouble scoring and felt they had to explain their scores. The original welfare framework was never devised to score instant death. One assessor said they “did not understand how to score the WATWC for a dead whale” and another said they assumed they could not score the welfare of a dead animal. Therefore, it was decided to include it separately for analysis.

### Event duration

The event duration scores varied considerably between assessors, especially amongst the ‘welfare experts.’ This potentially highlighted a lack of knowledge about collisions and cetacean injuries, Therefore, perhaps veterinarians or cetacean experts would be the more suitable to answer this section. This was also implied by a welfare expert who did not feel knowledgeable enough on whales to give an answer. One expert recommended that in the future, confidence in duration score should be noted (as well as confidence in D1–D5 scores). The wide variation in responses, and no uniformity to them, made event duration hard to assess. In the future, options should be given for assessors to choose from, for example, ‘seconds/minutes’, ‘days’, ‘weeks’, ‘months’, ‘years’ and ‘until death.’ However, an important point to note is that a Domain 5 harm of > 1 may persist for months to years in even the least severe cases, with ‘months’ being the modal D5 harm duration for Case 1A.

### Absolute versus relative preference

The hypothesis that participants would rate in a relative fashion was rejected in this study with no significant difference between the scores of assessors from Group A and Group B. This suggests scores for a given scenario may be comparable between studies, rather than influenced by the severity of accompanying cases presented to assessors. This was a novel approach to the WATWC and provides helpful information to future deployments. However, it should be tested again to confirm assessors score absolutely across a wider range of contexts.

### Feedback from assessors

The number of experts that took part in this study (n = 29) was considerably greater than that in the original deployment by Nicol *et al*. (2020), where 12 took part and in similar welfare assessments in other species, e.g. McGreevy *et al*. (2018), where 16 experts took part. The larger sample size allowed for more accurate means and to identify outliers.

It was suggested that the background information sheet needed more detail, including concerning the social complexity of wild cetaceans; their social group structure; ‘hierarchy’ formation; mutual help; and mother-offspring relations. One respondent suggested veterinarians who know the stress literature may provide a more expert view. However, currently, little stress literature exists on wild cetaceans.

The instructions and documents proved to be adequately self-explanatory. Three advised the survey should use one link rather than an Excel spreadsheet. However, this did not prove possible for this deployment due to the need to refer back to the cases, background information sheet and Five Domains model for wild cetaceans.

One assessor, Dr Ngaio Beausoleil (who agreed to having these comments attributed to her), recommended via personal communications:

- Domain 5 scores should be assigned as the highest impact from Domains 1–4;
- Confidence in event duration score should have been noted;
- It is important to differentiate between ‘welfare risks’ and ‘welfare impacts’ in designating scores;
- A ‘none’ to ‘extreme’ ranking system may be more beneficial (Beausoleil & Mellor 2015) because numerical data can encourage people to undertake unjustifiable calculations (e.g. averaging) and analyses that can lead to misleading outcomes. It also suggests precision that is not possible when evaluating inferred states.

Due to retrospective allocation of participants to the welfare expert or cetacean specialist grouping, there was a degree of confounding between expert background and Group. Five cetacean specialists and ten welfare experts received Case 1A and eleven cetacean specialists and three welfare experts received Case 1B. Although when analysed separately neither expert background nor the initial case received had a significant influence on scoring, the confound still needs to be acknowledged. Results may have differed had a higher proportion of welfare experts received the more severe Case 1B, for example.

### Future use of the WATWC

Future use would benefit from bringing the experts together into a post-survey discussion where expertise could be shared, as has been done in other welfare assessments using the Five Domains model (McGreevy *et al*. 2018). The knowledge gap between ‘welfare’ and ‘cetacean’ experts would be better bridged in this way and views on the cases shared to come to the most informed conclusion. Since animal welfare is a multi-disciplinary science (Dawkins 2008; Clegg & Butterworth 2017; Dolman *et al*. 2020; Nicol *et al*. 2020; Clegg *et al*. 2021), the sharing of expertise would be very useful. Expert scores could be assessed again after the post-survey discussion to assess whether the sharing of expertise had influenced their scoring.

A follow-up discussion between experts could not be organised for this study because of time constraints. A Zoom meeting could be arranged to continue this research which may reveal relative preference was a factor in experts’ decision-making; and for the case of the whale who died instantly, it was perhaps not as detrimental to welfare as some experts implied.

The need to apply such frameworks to these situations is likely to become increasingly important in the future as large-scale port developments increase the likelihood of ship-strikes, as do global increases in shipping (Tournadre 2014). New port developments are a growing worldwide phenomenon (for example, the planned major port development in Lamu, Kenya [LAPSSET 2017]). Where they are adjacent to important cetacean habitat or cause increased shipping to cross such habitat, including whale migration routes, they may lead to increased ship strikes (KMMREC 2020; Mwango-mbe *et al*. 2020). Some efforts to address this have been made by changing shipping routes and speeds; for example, across Cape Cod on the entry route to Boston Harbour, which cuts across the Stellwagen Bank Marine Reserve, an important area for whales (Wiley *et al*. 2011).

This study highlighted an information gap in ship-strike data which lead to limitations in information gathering for the cases used in this trial. More information was needed on: the outcome of the injuries and future of the individual (e.g. if it became infected and progressed to septicaemia or if the individual recovered); predation risk (e.g. whether a shark attacked the injured calf or whether the calf was protected by its mother until it healed/died); ship-traffic density and speed (both in their feeding ground and breeding ground, as well as on their migratory routes); and diagrams or photographs showing the extent of the injury. Future deployments could benefit from a qualified veterinarian with knowledge of cetacean injuries to aid the writing up of the case studies with suggested likely outcomes for the individuals concerned.

## Animal welfare implications and conclusion

With shipping activities showing an upward trend in our oceans, ship-strike is only going to become more of a threat to cetaceans. This study highlighted the severity of these collisions to whales’ welfare and is the first study to focus on the welfare consequences of ship-strike. Whales may suffer some level of Domain 5 harm (> 1) for the rest of their lives, even if the incident is not fatal. This confirms ship-strike is a welfare issue of significance, even if it does not impact population-viability. It should be added to legislation considerations especially in ‘high-risk’ areas where ships and whales converge, such as port approaches (such as that of Lamu Port in Kenya). The domain results from this study can be applied to other scenarios, such as any situation where a calf is orphaned, or entanglements involving tail damage. This is the first attempt to see whether assessors score on an absolute or relative scale. Being given Case 1A or Case 1B only had a small effect on assessors scores suggesting relativity does not play a part in assessor scoring. This study will aid future deployments of the WATWC. It highlighted the importance of bringing together experts to form a group conclusion and the necessity to include the outcome of the event in the case studies. Overall, the WATWC proved very useful for a quick and effective assessment and is a considerable advancement in the world of wild cetacean welfare.

## Supplementary material

For supplementary material accompanying this paper visit https://doi.org/10.1017/awf.2023.7. click here to view supplementary material
