# Supplementary material for: Expert assessment of the impact of ship-strikes on cetacean welfare using the Welfare Assessment Tool for Wild Cetaceans
Source: Anim Welf. 2023 Feb 23;32:e18. doi: 10.1017/awf.2023.7 (PMC10936308; doi:10.1017/awf.2023.7)
Supplement: Supplementary file 1 [file S0962728623000076sup001.pdf]

Supplementary Material

Participant Backgrounds (as stated by the experts)

- Zoo and wildlife vet
- Animal welfare science, animal behaviour science, animal welfare ethics, animal welfare assessment systems, conservation welfare science, neonatal and mother-young sciences, pain assessment and management, science-based welfare standards, slaughter science, wildlife welfare science
- Welfare specialist with interest in cetacean science
- Probably a generalist, some cetacean and welfare knowledge
- I would have no problem being called a welfare specialist. But if you had asked me initially to describe my expertise I would have said 'animal welfare scientist' and 'ethologist'.
- A cetacean specialist who places an emphasis on animal welfare
- Cetacean scientist
- Cetacean scientist
- Animal welfare biologist
- Welfare specialist

- “Cetacean expert with special knowledge (strandings) and focus on welfare
- Cetacean conservationist with a special interest in welfare
- Cetacean scientist
- I think I would classify myself as a cetacean conservationist. I oversee the research with the humpback whales (and collaborate on many different projects), but they are personally not my main focus (I am a shark and ray scientist). The research that I do with humpback whales is to help with their conservation, but I am not directly working with them personally.
- Cetacean scientist with experience in understanding and mitigating human impact (i.e. entanglement and ship strikes). And some exposure to ideas of welfare issues for wild cetaceans.
- Welfare specialist (mostly working with dairy cattle).
- Cetacean welfare scientist.
- I am a veterinarian in charge of the stranding network of cetaceans in Reunion Island, and a biologist. My expertise is focused on dolphins’ ecology (photo-identification, abundance, feeding habits, sociality) and on humpback whales (photoid, acoustic and habitat). I am also in charge of the welfare of cetaceans in the NGO I work for.
- I guess I would say my expertise would be in cetaceans. However, my PhD (which I am only a year into) is focussed around welfare science and cetaceans. But until my PhD my work focussed on marine mammals generally, so probably cetacean scientist.
- Welfare science specialist with specific expertise in the application of five domains model
